# Candidate proteomic indirect-effect signals linking dementia to probable muscle-bone fragility: a UK Biobank cohort study

**DOI:** 10.3389/fmed.2026.1883046

**Published:** 2026-09-09

**Authors:** Jiashu Yue, Jian Huang, Tongtong Zhang, Qianying Hao, Gang Liu, Huizhong Bai, Yu Jiang, Yunqiao Zhou, Jinyu Li, Xiaohong Mu

**Affiliations:** 1 Beijing University of Chinese Medicine Affiliated Dongzhimen Hospital, Beijing, China; 2 Guang'anmen Hospital, China Academy of Chinese Medical Sciences, Beijing, China; 3 State Key Laboratory of Tribology in Advanced Equipment, Department of Mechanical Engineering, Tsinghua University, Beijing, China

**Keywords:** circulating biomarkers, dementia, muscle–bone crosstalk, musculoskeletal fragility, plasma proteomics, probable osteosarcopenia proxy, UK Biobank

## Abstract

**Background:**

The systemic impact of dementia on musculoskeletal health remains poorly characterized. We investigated associations of algorithmically ascertained dementia status with bone fragility, functional trajectories, and low grip strength-defined probable sarcopenia, and explored candidate circulating proteomic indirect-effect signals for these associations.

**Methods:**

In a prospective UK Biobank cohort of 151,751 participants (median follow-up 15.1 years), dementia status was defined from algorithmically derived diagnosis records spanning linked follow-up. Descriptive fixed group-status models evaluated associations with incident osteoporosis and fracture, longitudinal grip-strength and physical-activity trajectories, and baseline probable sarcopenia and probable osteosarcopenia proxy. A time-updated Cox analysis assigned person-time before and on the recorded diagnosis date to the reference state and person-time strictly after that date to the exposed state. In a proteomic subcohort (*n* = 16,652; Olink Explore 3,072), two-stage screening of 2,923 proteins identified candidate protein signals; these were then evaluated using exploratory, model-based indirect-effect analyses with bootstrap resampling.

**Results:**

In time-updated models, all-cause dementia after diagnosis was associated with osteoporosis (HR = 1.78, 95% CI 1.43–2.20) and fracture (HR = 3.68, 95% CI 2.34–5.77), with estimates strongest within 0–2 years after diagnosis and attenuated thereafter. For descriptive context, fixed group-status analyses of recorded dementia ascertainment status showed associations with osteoporosis (HR = 2.31, 95% CI 2.06–2.59) and fracture (HR = 3.13, 95% CI 2.27–4.31), accelerated grip decline (*β* = −0.317 kg/year, *p* = 1.3 × 10^−5^), and higher baseline odds of probable sarcopenia (OR = 1.41, 95% CI 1.23–1.61). Under the prespecified observed-sample criteria, EGFR (proportion mediated [PM] = 8.7%), EBI3/IL27 (PM = 5.9%), and CKB (PM = 3.8%) had modest indirect-effect estimates. Across the age-adjusted first-stage screen and the 500-resample full-pipeline bootstrap, EGFR and EBI3/IL27 were the most consistently supported signals; CKB showed lower joint selection stability and was classified as exploratory.

**Conclusion:**

In the UK Biobank, algorithmically ascertained dementia status was associated with bone fragility, functional trajectories, and baseline probable sarcopenia-related phenotypes. Time-updated analyses supported elevated postdiagnosis bone risks, with the strongest estimates near the time of diagnosis. EGFR and EBI3/IL27 were the most consistently supported candidate circulating indirect-effect signals for the recorded dementia status–probable sarcopenia association. CKB met the prespecified observed-sample criteria but showed lower selection stability and was retained as exploratory; individual proportions mediated were modest.

## 1 Introduction

Dementia affects approximately 55 million people worldwide, with projections exceeding 150 million by 2050 (1, 2). Yet while its neurological consequences are well studied, the systemic effects on the musculoskeletal system remain poorly understood (3, 4). Clinically, dementia patients experience more fractures, faster functional decline, and greater frailty, but the pathways connecting central neurodegeneration to peripheral musculoskeletal damage are not well defined. This gap matters clinically: musculoskeletal complications, including fractures and sarcopenia, are major drivers of institutionalization, mortality, and loss of independence in older adults with dementia (5–8).

Prior epidemiological studies have documented individual components of this picture. Observational studies have reported elevated fracture risk in dementia, and hip fracture in particular is associated with prolonged disability and loss of independence in older adults (9–11). Cross-sectional analyses have linked sarcopenia to increased osteoporosis and fracture risk (12–14), while cognitive impairment has been associated with low grip strength and reduced appendicular lean mass. However, most existing work has examined single outcomes in isolation, relied on relatively small or clinically selected samples, or lacked longitudinal follow-up sufficient to resolve temporal ordering between dementia onset and musculoskeletal deterioration. Bone fragility, functional decline, and sarcopenia phenotypes have rarely been characterized together at population scale within a single analytical framework.

These associations likely span multiple domains, reflecting the broad systemic reach of dementia-related pathology and shared aging-related processes. At the skeletal level, immobility, vitamin D deficiency, and neuroendocrine disruption may accelerate bone loss (15, 16), increasing susceptibility to osteoporosis and fracture. At the muscular level, impaired motor unit recruitment, reduced physical activity, and neuroinflammatory spillover may promote functional decline and probable sarcopenia (17), often preceding measurable losses in muscle mass, consistent with the EWGSOP2 (European Working Group on Sarcopenia in Older People 2) framework (18). These mechanisms may converge with vascular disease, chronic inflammation, frailty, nutrition, medication use, and socioeconomic factors to produce concurrent bone fragility, functional decline, and probable sarcopenia-related phenotypes in dementia. Whether these associations reflect shared risk factors, dementia-related processes, or candidate molecular links remains to be systematically established in large prospective cohorts.

Population-scale plasma proteomics now enables systematic investigation of candidate molecular links between chronic diseases and systemic phenotypes (19–21). High-throughput platforms such as Olink Explore simultaneously quantify thousands of circulating proteins with high specificity, providing broader coverage of inter-organ signaling than conventional candidate-biomarker approaches. Screening thousands of circulating proteins for associations with both exposure and outcome can prioritize proteins for exploratory model-based indirect-effect analysis. Prior work has applied plasma proteomics to identify protein signals linking degenerative joint disease and dementia risk (22), but the reciprocal dementia-to-musculoskeletal-vulnerability association remains less well characterized.

We used UK Biobank data to investigate: (i) whether algorithmically ascertained dementia status was associated with incident osteoporosis and fracture, longitudinal functional trajectories, and baseline sarcopenia-related phenotypes; and (ii) whether circulating proteins, identified via systematic Olink proteomic screening, showed exploratory model-based indirect-effect signals for the recorded dementia status–probable sarcopenia association, with probable sarcopenia selected as the primary proteomic endpoint because handgrip strength is a clinically relevant functional marker (23) and the baseline phenotype definition afforded adequate statistical power in the proteomic subcohort.

## 2 Methods

### 2.1 Study population and design

This study used data from the UK Biobank, a prospective population-based cohort of approximately 500,000 participants aged 37–73 years at recruitment (2006–2010). We applied a multistage design spanning epidemiological, longitudinal, and proteomic analyses to characterize dementia-associated bone fragility, functional decline, and probable sarcopenia-related phenotypes.

The local UK Biobank source population contained 501,936 participants. The main epidemiological Part 1 source cohort comprised 154,056 participants with eligible baseline clinical and bone data. For time-to-event analyses of incident osteoporosis and osteoporotic fracture, we excluded 2,305 participants whose follow-up time was zero years or less. All 2,305 participants in this exclusion group had osteoporosis first-occurrence dates on or before baseline. The final time-to-event analytic cohort therefore included 151,751 participants. Mean age at baseline was 55.9 ± 8.1 years; 69,966 (46.1%) were women and 81,785 (53.9%) were men. The median follow-up was 15.1 years (interquartile range [IQR] 14.7–15.5), accumulating 2,251,779 person-years of observation. During follow-up, 4,776 incident osteoporosis events and 408 incident osteoporotic fracture events were documented.

Dementia ascertainment used UK Biobank algorithmically derived diagnosis-date fields spanning linked follow-up. All-cause dementia (ACD), Alzheimer’s disease (AD), and vascular dementia (VaD) indicators were defined by non-missing p42018, p42020, and p42022 dates, respectively. Fields p42024, p130836, and p131036 were inspected as supporting linked subtype records but did not define or override the primary ACD, AD, or VaD indicators. The primary indicators were modeled separately, and AD and VaD were not mutually exclusive. In the Part 1 source cohort before excluding non-positive follow-up time, there were 3,236 ACD, 1,429 AD, and 748 VaD records. In the final time-to-event cohort, the corresponding counts were 3,146, 1,387, and 728. Of these, 50 ACD, 2 AD, and 4 VaD diagnosis dates occurred on or before baseline; 3,096 ACD, 1,385 AD, and 724 VaD dates occurred after baseline. There were 163 participants meeting both AD and VaD indicators, and 1,194 ACD participants had neither an AD nor a VaD indicator. The complete Boolean definitions, diagnostic timing, overlap counts, and date-boundary handling are reported in Supplementary Tables S16A,D–E.

For baseline probable sarcopenia-related bridge phenotypes, eligibility was based on availability of handgrip strength and heel eBMD, while positive follow-up time was required for incident bone outcomes. This phenotype-ready bridge cohort retained 153,994 participants. For the proteomic analyses, 52,995 participants had Olink Explore 3,072 plasma proteomic data in the local extraction. The overlap with the Part 1 source table contained 16,661 participants, and the phenotype-ready Olink subcohort contained 16,652 participants after requiring the probable sarcopenia definition. The UKB-PPP Olink resource includes random baseline and additional non-random selection components; sampling weights were unavailable in the current analysis-ready files, so proteomic analyses were conducted as unweighted models. We therefore report included-versus-excluded comparisons and standardized mean differences to characterize possible selection.

### 2.2 Outcomes

Primary clinical outcomes were incident osteoporosis (ICD-10: M80 or M81) and incident osteoporotic fracture (ICD-10: M80, osteoporosis with current pathological fracture), ascertained through linked hospital inpatient records. Because M80 is nested within the broader osteoporosis definition, osteoporosis and osteoporotic fracture were analyzed and interpreted as related but distinct bone-fragility endpoints. Participants with osteoporosis first-occurrence dates on or before baseline were excluded from time-to-event analyses through the non-positive follow-up filter described above; among these 2,305 excluded participants, 115 had M80 dates on or before baseline and 2,221 had M81 dates on or before baseline. These code-specific counts were not mutually exclusive because some participants had both M80 and M81 dates.

Functional outcomes were assessed through longitudinal changes in hand grip strength (UK Biobank fields 46/47) and total physical activity (field 924) across repeated assessment visits. To bridge the main-cohort epidemiological findings and the Olink-based proteomic analyses, we defined two baseline composite phenotypes in the full cohort: probable sarcopenia, defined as maximum handgrip strength < 27 kg in men or < 16 kg in women (fields 46/47; EWGSOP2 low-grip criterion), using the higher value from either hand; and probable osteosarcopenia proxy, defined as concurrent probable sarcopenia plus sex-specific lowest-quartile heel estimated bone mineral density (eBMD; field 3,148). Confirmed sarcopenia requires muscle quantity or quality assessment; incomplete lean-mass availability limited confirmed-sarcopenia evaluation in the full cohort. We therefore evaluated confirmed sarcopenia as an available-subset descriptive check where muscle-mass data were present.

Grip strength may be affected by comprehension, motivation, coordination, pain, arthritis, and testing cooperation, particularly among participants with cognitive impairment. Therefore, all low-grip outcomes are described as low grip strength-defined probable sarcopenia, and the composite muscle-bone phenotype is described as a probable osteosarcopenia proxy.

### 2.3 Epidemiological analyses (part 1)

#### 2.3.1 Cox proportional hazards models

For incident osteoporosis and fracture, we fitted multivariable Cox proportional hazards models with a hierarchical adjustment strategy. In these fixed group-status models, dementia indicators represented whether an algorithmic diagnosis was recorded across linked follow-up. Model 1 included age and sex as a reference adjustment model. Model 2 additionally adjusted for body mass index (BMI), standing height, and physical activity as a main clinical adjustment model (24, 25). Model 3 further incorporated 11 circulating biomarkers (vitamin D, calcium, phosphate, alkaline phosphatase, insulin-like growth factor-1, testosterone, C-reactive protein, albumin, creatinine, cystatin C, and glycated hemoglobin [HbA1c]). Because physical activity and several biomarkers may partly reflect downstream consequences of dementia or disease severity, Model 3 was interpreted as an extended conditional association model. Hazard ratios (HRs) with 95% confidence intervals (CIs) were reported across models.

#### 2.3.2 Sensitivity analyses

Four sensitivity analyses were performed for the bone-fragility outcomes. First, Fine–Gray subdistribution hazard models were used to account for death as a competing event. Second, propensity score matching (PSM) using 1:3 nearest-neighbor matching with a caliper width of 0.1 standard deviations of the logit of the propensity score was used to balance baseline covariates; Cox models were then refit in the matched sample. Third, a 2-year baseline lag analysis excluded participants with follow-up ≤2 years and events occurring within 2 years of baseline. Fourth, counting-process Cox models treated dementia status as time updated: person-time before and on the recorded diagnosis date contributed to the reference state, and person-time strictly after that date was divided into two postdiagnosis exposure windows: 0–2 years and more than 2 years. Because diagnosis and outcome dates had day-level resolution, same-day diagnosis-outcome events were assigned to the reference interval when within-day ordering was unknown. The time-updated models used the same 151,751-participant cohort, outcome definitions, and Model 3 covariates as the fixed group-status analyses (Supplementary Tables S16B–E).

To address additional sociodemographic and lifestyle confounding, we fitted an additional sensitivity model adjusted for age, sex, BMI, height, ethnicity, Townsend deprivation quartile, education, alcohol consumption, smoking status, hypertension, cardiovascular disease, and diabetes. This confounder-oriented model omitted physical activity and circulating biomarkers to reduce conditioning on variables that may lie on the dementia-to-musculoskeletal pathway. All models were interpreted as adjusted association models, without claiming identification of a strict causal total effect. Missing or blank education entries were retained as a missing/unclassified category; low-frequency missingness in Townsend deprivation, alcohol consumption, smoking status, and diabetes was handled by model-specific complete-case analysis.

#### 2.3.3 Longitudinal functional decline

Linear mixed-effects models (LMMs) with random intercepts and slopes were used to compare annual grip-strength and physical-activity trajectories according to dementia ascertainment status. A time × dementia-status interaction term captured the differential rate of change. These models describe trajectories among participants grouped by whether dementia was recorded during linked follow-up and do not isolate postdiagnosis change. Models were adjusted using the same covariate structure as Model 3 described above. Because repeat UK Biobank assessments were available only for selected returners, we report the number and percentage with repeat measures, measurement counts, timing of last follow-up, deaths, repeat-versus-no-repeat comparisons, and model-specific observation counts. Linear time trajectories were specified because contributing participants provided an average of approximately 2.3 grip-strength assessments and 2.2 physical-activity assessments; random intercepts and slopes allowed between-participant variation in baseline level and rate of change. Attendance summaries were based on participants in the Part 1 longitudinal source files with at least one valid outcome measurement; LMM samples required repeat measurements and complete model-specific variables, whereas repeat-versus-no-repeat comparisons were restricted to the 151,751-participant time-to-event cohort (Supplementary Tables S13A–C).

#### 2.3.4 Bridge phenotype analyses

To establish the relationship between dementia ascertainment status and the baseline sarcopenia-related outcomes used in downstream proteomic analyses, we fitted logistic regression models for probable sarcopenia and probable osteosarcopenia proxy with the same three-level covariate structure as the main association analyses. These baseline phenotype models were interpreted without temporal ordering.

### 2.4 Proteomic mediation analyses (part 2)

#### 2.4.1 Two-stage protein screening

We employed a two-stage screening strategy across 2,923 Olink proteins. In the primary Stage 1 screen, multivariable linear regression models adjusted for sex, BMI, and height tested the association between dementia exposure and each rank-based inverse-normal-transformed protein level. We also reran Stage 1 with age added to the sex-, BMI-, and height-adjusted model. In Stage 2, logistic regression models adjusted for 13 covariates (age, sex, BMI, height, ethnicity, Townsend deprivation quartile, education, smoking, alcohol, physical activity category, hypertension, diabetes, and cardiovascular disease) tested each protein’s association with probable sarcopenia, probable osteosarcopenia proxy, and the primary binary incident-osteoporosis indicator. Proteins passing both stages at Benjamini-Hochberg false discovery rate (FDR) < 0.01 were retained for the primary cross-layer overlap analysis. The primary cross-layer overlap yielded 276 proteins for probable sarcopenia, 228 for probable osteosarcopenia proxy, and 11 for incident osteoporosis, with five proteins supported across all three layers: GDF15, EGFR, EBI3/IL27, CKB, and CHGA.

Because incident osteoporosis is a time-to-event outcome, we reran the incident-osteoporosis screen for the exact primary family of 600 proteins that met BH-FDR < 0.01 in the ACD-to-protein Stage 1 analysis. We also evaluated the extended 706-protein overlap family, defined by at least one ACD/AD/VaD-to-protein association and at least one eBMD/DXA-ALM association at BH-FDR < 0.05 on both sides. The two families shared 559 proteins and contained 747 unique proteins in total; family-specific BH correction was applied separately across the 600 and 706 Cox *p* values (Supplementary Tables S9C,D). Detailed event counts and Schoenfeld-residual proportional-hazards tests from the same full 13-covariate Cox models were reported for the five prioritized proteins (Supplementary Table S9A), and Fine-Gray models treating death as a competing event were fitted for these five proteins as a sensitivity analysis (Supplementary Table S9B).

#### 2.4.2 Exploratory model-based mediation analysis

Exploratory model-based mediation analysis was conducted for the five prioritized triple-supported proteins using the R mediation package with nonparametric bootstrap (1,000 iterations). Because both circulating proteins and sarcopenia-related outcomes were measured at baseline in the Olink subcohort, these analyses were interpreted as exploratory indirect-effect analyses within an observational framework. The mediator model was a linear regression predicting rank-transformed protein level from dementia exposure and the 13-covariate set. The outcome model was a logistic regression predicting the binary probable sarcopenia-related outcome from dementia exposure, protein level, and the same covariate set. ACME estimates were reported following the terminology of the mediation package and interpreted as model-based indirect-effect estimates. Proteins with FDR-significant ACME (FDR < 0.05) and a significant total effect were classified as statistically supported candidate indirect-effect signals. The remaining triple-supported proteins were retained as exploratory signals in the mediation display to preserve biological context. Protein-specific analytic sample sizes varied because of per-protein missingness in Olink measurements and complete-case covariate availability. To evaluate whether the large proportion of missing education data influenced the mediation findings, we reran the same binary mediation models after excluding education from the covariate set, with the full comparison reported in Supplementary Table S7.

To quantify uncertainty arising from protein selection, we performed a selection-aware, participant-level bootstrap with 500 resamples (seed 20,260,801). In each resample, we repeated the age-, sex-, BMI-, and height-adjusted Stage 1 screen across the available Olink proteins, repeated the 13-covariate Stage 2 probable-sarcopenia screen, and refitted the indirect-effect models for the five prioritized proteins. BH-FDR < 0.01 was applied at both screening stages. We report Stage 1 and joint selection frequencies, fixed-target refit intervals across all resamples, and zero-augmented full-pipeline summaries in which the indirect-effect estimate was set to zero when a target protein failed joint selection (Supplementary Table S15). The fixed-target intervals quantify effect-estimate variability while holding the selected five-protein target set fixed and therefore remain susceptible to post-selection bias. The zero-augmented distribution combines effect magnitude with selection stability; exact zeros arise by construction in resamples where a target fails joint selection.

Because the same Olink subcohort was used for protein screening and downstream exploratory analyses, the prioritized proteins remain susceptible to winner’s-curse effects and post-selection bias. The full-pipeline bootstrap propagates internal selection uncertainty from both screening stages through indirect-effect estimation within the available UK Biobank Olink subcohort. It does not provide external validation, so the results remain hypothesis-generating candidate indirect-effect signals requiring independent replication. Individual proportions mediated were estimated in separate single-protein models and reported as individual model-based estimates. Independent replication, cis-pQTL colocalization, and Mendelian randomization are needed to triangulate these signals where suitable instruments are available.

#### 2.4.3 Dose–response modeling

Restricted cubic spline (RCS) logistic regression was used to evaluate the dose–response relationships of EGFR, EBI3/IL27, and CKB with probable sarcopenia. Models adjusted for dementia exposure and the full covariate set. Knots were placed at the 5th, 35th, 65th, and 95th percentiles of the standardized protein distribution. Both overall association and nonlinearity were tested. Per-standard-deviation Cox proportional hazards models, adjusted for the same 13-covariate set, were fitted to quantify the association between each protein and incident osteoporosis in the Olink subcohort.

### 2.5 Statistical software

All analyses were performed in R (version 4.2.2), using the following packages: survival and survminer (survival and cumulative-hazard analyses), cmprsk (Fine-Gray competing-risk models), MatchIt (propensity score matching), lme4 (linear mixed-effects models), mediation (model-based indirect-effect analysis), and rms (restricted cubic spline regression). This study was reported in accordance with the STROBE guidelines for observational epidemiological studies. A two-sided *p* value < 0.05 was considered statistically significant for epidemiological analyses. FDR-adjusted *p* values < 0.01 were used for proteomic screening, and FDR-adjusted ACME *p* values < 0.05 were used for exploratory model-based mediation analyses.

## 3 Results

### 3.1 Study population and baseline characteristics

Of 154,056 UK Biobank participants with eligible baseline data, 151,751 were retained in the Part 1 analytic cohort after excluding 2,305 individuals with non-positive follow-up (Table 1). This cohort included 3,146 participants with an algorithmically recorded all-cause dementia diagnosis across linked follow-up; 50 diagnosis dates occurred on or before baseline and 3,096 occurred after baseline. The median follow-up was 15.09 years (interquartile range 14.68–15.50), during which 4,776 incident osteoporosis events and 408 incident osteoporotic fracture events were recorded. In parallel, the phenotype-ready Olink subcohort comprised 16,652 participants, including 521 with recorded all-cause dementia status, 689 with probable sarcopenia, and 207 with probable osteosarcopenia proxy; its baseline characteristics are summarized in Supplementary Table S1.

**Table 1:** Baseline characteristics according to dementia ascertainment status across linked follow-up.

Characteristic	No recorded ACD	Recorded ACD		Recorded AD		Recorded VaD		SMD
	*N* = 148,605	*N* = 3,146	*p* value	*N* = 1,387	*p* value	*N* = 728	*p* value	
Cohort size and continuous variables								
Participants, *n*	148,605	3,146		1,387		728		
Age, years	55.65 (8.08)	64.15 (4.76)	< 0.001	64.46 (4.44)	< 0.001	64.83 (4.14)	< 0.001	1.281
BMI, kg/m^2^	27.38 (4.62)	27.73 (4.56)	< 0.001	27.38 (4.32)	0.986	28.39 (4.62)	< 0.001	0.076
Height, cm	169.99 (9.19)	169.15 (8.96)	< 0.001	168.79 (9.21)	< 0.001	169.25 (8.28)	0.016	0.093
Heel eBMD, g/cm^2^	0.553 (0.139)	0.542 (0.151)	< 0.001	0.543 (0.148)	0.010	0.536 (0.161)	0.005	0.074
Grip strength, kg	34.98 (11.36)	32.70 (10.65)	< 0.001	32.47 (10.70)	< 0.001	32.83 (10.16)	< 0.001	0.207
Follow-up, years, median (IQR)	15.09 (14.68–15.50)	15.08 (14.62–15.50)	0.002	15.08 (14.62–15.52)	0.058	15.10 (14.63–15.48)	0.095	0.204
Demographic and socioeconomic variables								
Sex, *n* (%)			< 0.001		< 0.001		< 0.001	0.23
Female	68,861 (46.3%)	1,105 (35.1%)		539 (38.9%)		215 (29.5%)		
Male	79,744 (53.7%)	2,041 (64.9%)		848 (61.1%)		513 (70.5%)		
Ethnicity, *n* (%)			0.006		0.250		0.042	0.054
White	143,885 (96.8%)	3,074 (97.7%)		1,351 (97.4%)		715 (98.2%)		
Non-white	4,720 (3.2%)	72 (2.3%)		36 (2.6%)		13 (1.8%)		
Townsend deprivation quartile, *n* (%)			0.003		0.911		0.022	0.069
Q1 (least deprived)	37,481 (25.2%)	706 (22.4%)		344 (24.8%)		150 (20.6%)		
Q2	36,571 (24.6%)	783 (24.9%)		336 (24.2%)		178 (24.5%)		
Q3	36,530 (24.6%)	794 (25.2%)		352 (25.4%)		194 (26.6%)		
Q4 (most deprived)	37,884 (25.5%)	860 (27.3%)		355 (25.6%)		206 (28.3%)		
Education, *n* (%)			< 0.001		< 0.001		< 0.001	0.093
College/University	17,895 (12.0%)	296 (9.4%)		132 (9.5%)		67 (9.2%)		
A-levels	4,041 (2.7%)	78 (2.5%)		26 (1.9%)		16 (2.2%)		
O-levels/GCSE	16,281 (11.0%)	385 (12.2%)		159 (11.5%)		94 (12.9%)		
Other qualifications	21,467 (14.4%)	482 (15.3%)		216 (15.6%)		118 (16.2%)		
No qualifications	20,292 (13.7%)	981 (31.2%)		454 (32.7%)		232 (31.9%)		
Lifestyle variables								
Alcohol consumption, *n* (%)			< 0.001		< 0.001		< 0.001	0.179
Never	9,251 (6.2%)	348 (11.1%)		149 (10.7%)		86 (11.8%)		
Occasional	29,945 (20.2%)	657 (20.9%)		281 (20.3%)		150 (20.6%)		
Frequent	109,356 (73.6%)	2,139 (68.0%)		957 (69.0%)		492 (67.6%)		
Smoking status, *n* (%)			< 0.001		< 0.001		< 0.001	0.212
Never	81,231 (54.7%)	1,412 (44.9%)		645 (46.5%)		296 (40.7%)		
Previous	51,788 (34.8%)	1,412 (44.9%)		620 (44.7%)		336 (46.2%)		
Current	15,243 (10.3%)	310 (9.9%)		114 (8.2%)		94 (12.9%)		
Physical activity category, *n* (%)			0.006		< 0.001		0.148	0.058
Low	49,443 (33.3%)	1,070 (34.0%)		413 (29.8%)		264 (36.3%)		
Medium	49,675 (33.4%)	969 (30.8%)		428 (30.9%)		222 (30.5%)		
High	49,487 (33.3%)	1,107 (35.2%)		546 (39.4%)		242 (33.2%)		
Clinical variables and outcomes								
Hypertension, *n* (%)	37,028 (24.9%)	1,312 (41.7%)	< 0.001	503 (36.3%)	< 0.001	384 (52.7%)	< 0.001	0.362
Diabetes, *n* (%)	6,866 (4.6%)	408 (13.0%)	< 0.001	144 (10.4%)	< 0.001	154 (21.2%)	< 0.001	0.298
Cardiovascular disease, *n* (%)	7,716 (5.2%)	506 (16.1%)	< 0.001	172 (12.4%)	< 0.001	173 (23.8%)	< 0.001	0.359
Incident osteoporosis, *n* (%)	4,446 (3.0%)	330 (10.5%)	< 0.001	143 (10.3%)	< 0.001	84 (11.5%)	< 0.001	0.302
Incident osteoporotic fracture, *n* (%)	364 (0.2%)	44 (1.4%)	< 0.001	18 (1.3%)	< 0.001	17 (2.3%)	< 0.001	0.128

The STROBE-style cohort flow is shown in Supplementary Figure S1, Supplementary Tables S11A–C. The local source population contained 501,936 participants, of whom 154,056 formed the Part 1 source cohort with eligible baseline clinical and bone data. The time-to-event cohort contained 151,751 participants after excluding 2,305 participants with non-positive follow-up time; all 2,305 had osteoporosis first-occurrence dates on or before baseline. The phenotype-ready bridge cohort contained 153,994 participants for probable sarcopenia-related baseline phenotypes. The Olink phenotype-ready subcohort contained 16,652 participants.

In the Olink Cox-overlap source table, education was missing or unclassified for 7,538 of 16,661 participants (45.2%). Townsend deprivation, alcohol consumption, and smoking had low missingness (13, 7, and 37 missing values, respectively; Supplementary Table S12A). Olink-included and Olink-excluded participants were broadly similar for age, sex, BMI, height, and heel eBMD, with small standardized mean differences; the proportion with recorded dementia ascertainment status and mortality were modestly higher in the Olink-included group (Supplementary Tables S12B,C).

Participants with an algorithmically recorded dementia diagnosis during linked follow-up were older at baseline and had lower baseline grip strength and heel eBMD than participants without such a record in the full analytic cohort (Table 1), with a similar descriptive pattern in the Olink subcohort (Supplementary Table S1). These differences motivated the multilevel analysis structure used in the study.

Values are presented as mean (SD) or *n* (%) unless otherwise indicated. Follow-up time is presented as median (IQR). Dementia groups represent algorithmic ascertainment status across linked follow-up; AD and VaD indicators may overlap. SMD denotes the standardized mean difference for recorded ACD versus no recorded ACD. Education category was missing or unclassified for 69,553 participants (45.8%). Education was modeled as a categorical covariate in proteomic models, with blank or unclassified entries retained as a separate missing/unclassified category to preserve sample size and reduce selection caused by excluding participants solely because of missing education information. Sensitivity analyses excluding education from the covariate set were performed for the proteomic mediation models and summarized in Supplementary Table S7. Missingness was low for Townsend deprivation quartile (*n* = 142), alcohol consumption (*n* = 55), and smoking status (*n* = 355). ACD, all-cause dementia; AD, Alzheimer’s disease; VaD, vascular dementia; BMI, body mass index; eBMD, estimated bone mineral density; IQR, interquartile range.

### 3.2 Recorded dementia status was associated with elevated bone-fragility risk

**Figure 1:**
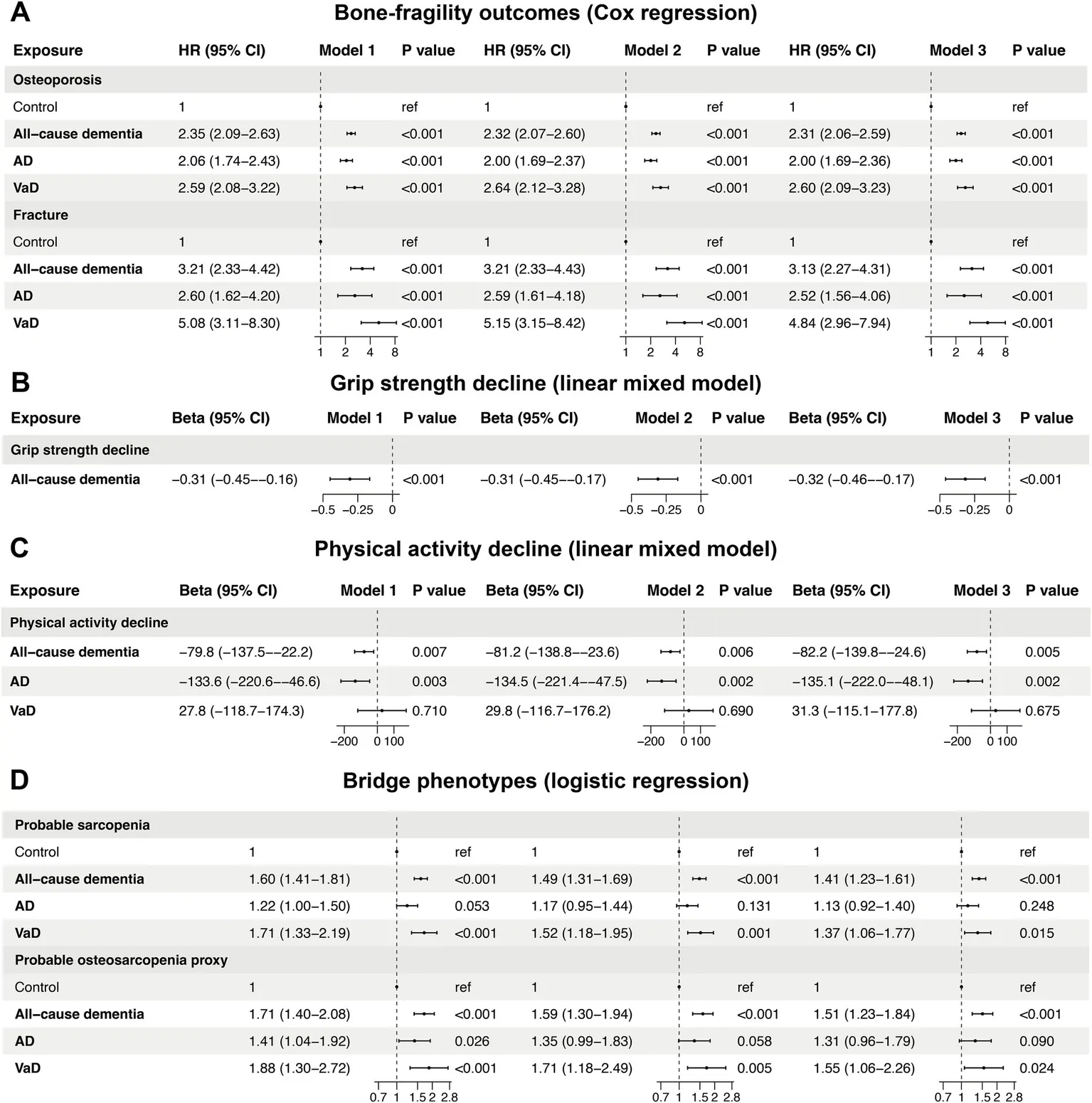
Epidemiological associations between dementia and musculoskeletal outcomes. **(A)** Forest plots showing fixed group-status HRs and 95% CIs for incident osteoporosis and osteoporotic fracture from Model 3. **(B)** Forest plot showing the adjusted linear mixed-effects estimate for the difference in annual grip-strength trajectory according to all-cause dementia ascertainment status. **(C)** Forest plot showing the corresponding physical-activity trajectory estimate. **(D)** Forest plots showing baseline ORs and 95% CIs for probable sarcopenia and probable osteosarcopenia proxy according to dementia ascertainment status. ACD, all-cause dementia; AD, Alzheimer’s disease; VaD, vascular dementia.

In fixed group-status models, recorded all-cause dementia status was associated with incident osteoporosis (HR = 2.31, 95% CI 2.06–2.59; *p* = 9.0 × 10^−47^) and osteoporotic fracture (HR = 3.13, 95% CI 2.27–4.31; *p* = 4.0 × 10^−12^) in Model 3 (Figure 1A; Table 2).

**Table 2:** Fixed group-status associations of algorithmically ascertained dementia with incident osteoporosis and osteoporotic fracture.

Analysis	Exposure	Outcome	Effect estimate	*p* value	Sample size	
			HR/SHR (95% CI)		*N*	Events
Primary fully adjusted Cox models						
Model 3	ACD	Osteoporosis	HR = 2.31 (2.06–2.59)	9.0 × 10^−47^	151,751	4,776
Model 3	AD	Osteoporosis	HR = 2.00 (1.69–2.36)	7.3 × 10^−16^	151,751	4,776
Model 3	VaD	Osteoporosis	HR = 2.60 (2.09–3.23)	7.0 × 10^−18^	151,751	4,776
Model 3	ACD	Osteoporotic fracture	HR = 3.13 (2.27–4.31)	4.0 × 10^−12^	151,751	408
Model 3	AD	Osteoporotic fracture	HR = 2.52 (1.56–4.06)	1.5 × 10^−4^	151,751	408
Model 3	VaD	Osteoporotic fracture	HR = 4.84 (2.96–7.94)	3.9 × 10^−10^	151,751	408
Sensitivity analyses						
Fine-Gray	ACD	Osteoporosis	SHR = 2.28 (2.03–2.56)	< 0.001	151,751	4,776
Fine-Gray	ACD	Osteoporotic fracture	SHR = 3.00 (2.15–4.19)	< 0.001	151,751	408
PSM	ACD	Osteoporosis	HR = 2.30 (1.99–2.66)	1.4 × 10^−29^	12,584	758
PSM	ACD	Osteoporotic fracture	HR = 2.70 (1.79–4.08)	2.2 × 10^−6^	12,584	92
2-year lag	ACD	Osteoporosis	HR = 2.40 (2.14–2.70)	6.4 × 10^−49^	151,443	4,468
2-year lag	AD	Osteoporosis	HR = 2.07 (1.74–2.45)	1.3 × 10^−16^	151,443	4,468
2-year lag	VaD	Osteoporosis	HR = 2.75 (2.20–3.42)	2.6 × 10^−19^	151,443	4,468
2-year lag	ACD	Osteoporotic fracture	HR = 3.21 (2.32–4.45)	2.6 × 10^−12^	151,443	387
2-year lag	AD	Osteoporotic fracture	HR = 2.64 (1.63–4.26)	7.2 × 10^−5^	151,443	387
2-year lag	VaD	Osteoporotic fracture	HR = 4.77 (2.87–7.93)	1.8 × 10^−9^	151,443	387

Model 3 was adjusted for age, sex, body mass index, height, physical activity, and 11 circulating biomarkers. Fine-Gray models treated death as a competing event. The matched sample used 1:3 nearest-neighbor propensity score matching. ACD, all-cause dementia; AD, Alzheimer’s disease; VaD, vascular dementia; HR, hazard ratio; SHR, subdistribution hazard ratio; CI, confidence interval.

The fixed group-status subtype analyses were directionally consistent. Recorded Alzheimer’s disease status was associated with osteoporosis (HR = 2.00, 95% CI 1.69–2.36; *p* = 7.3 × 10^−16^) and osteoporotic fracture (HR = 2.52, 95% CI 1.56–4.06; *p* = 1.5 × 10^−4^). Recorded vascular dementia status showed the numerically largest estimates, with HR = 2.60 (95% CI 2.09–3.23; *p* = 7.0 × 10^−18^) for osteoporosis and HR = 4.84 (95% CI 2.96–7.94; *p* = 3.9 × 10^−10^) for osteoporotic fracture. A VaD-focused cross-domain summary is provided in Supplementary Table S2.

### 3.3 Sensitivity analyses for bone-fragility outcomes

The bone-fragility associations persisted across three sensitivity analyses (Table 2). In Fine–Gray competing-risk models, the subdistribution hazard ratios were SHR = 2.28 (95% CI 2.03–2.56) for osteoporosis and SHR = 3.00 (95% CI 2.15–4.19) for osteoporotic fracture. In the 1:3 propensity score-matched sample, the corresponding HR = 2.30 (95% CI 1.99–2.66; *p* = 1.4 × 10^−29^) and HR = 2.70 (95% CI 1.79–4.08; *p* = 2.2 × 10^−6^), respectively.

**Figure 2:**
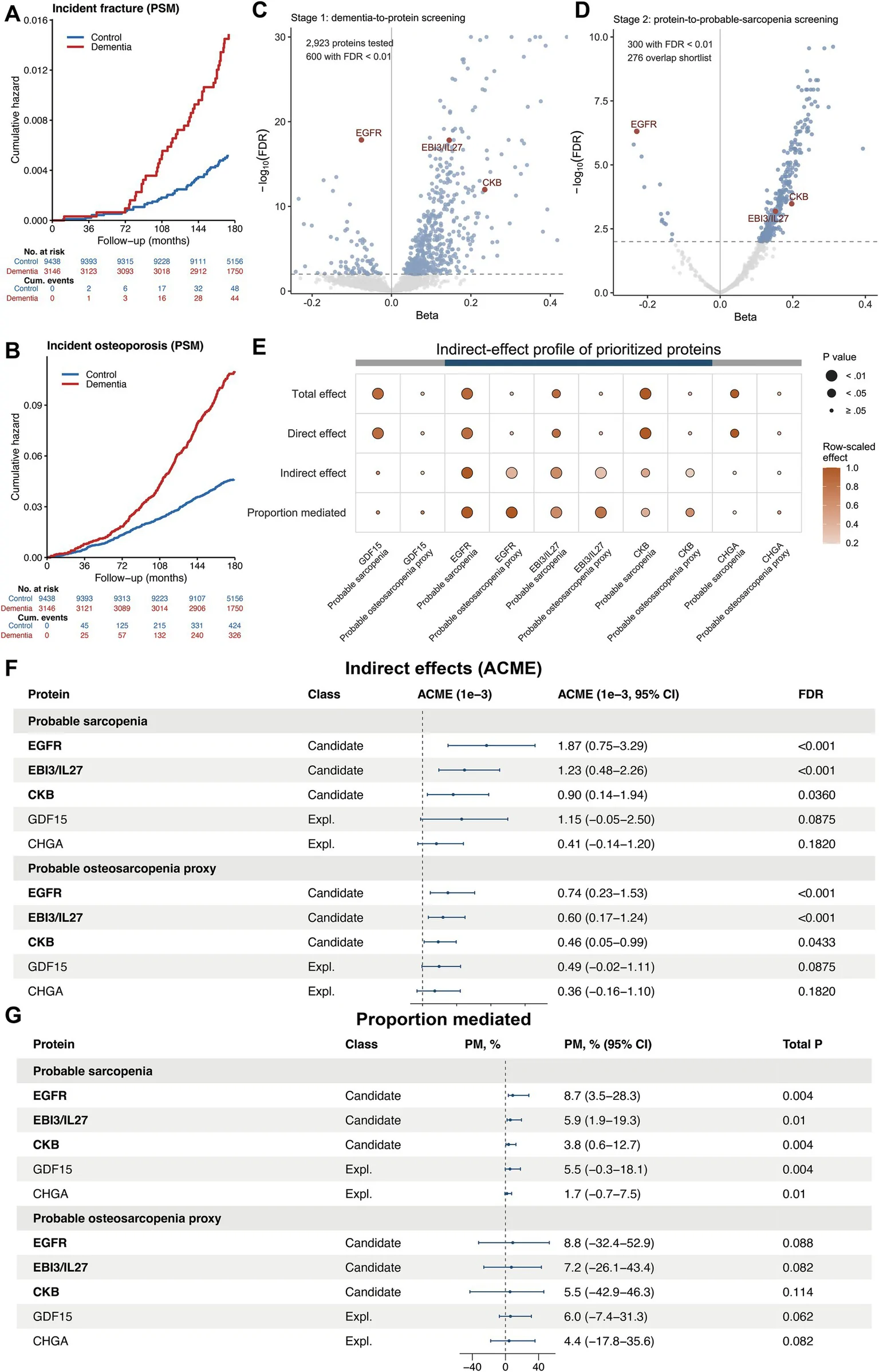
Propensity score-matched analyses, protein screening, and exploratory model-based mediation. **(A)** Cumulative hazard curves for incident osteoporotic fracture in the 1:3 propensity score-matched sample. **(B)** Cumulative hazard curves for incident osteoporosis in the matched sample. Blue lines denote controls; red lines denote participants with recorded all-cause dementia status. Numbers at risk and cumulative events are shown below each panel. **(C)** Stage 1 volcano plot showing dementia-status-to-protein screening results across 2,923 Olink proteins. **(D)** Stage 2 volcano plot showing protein-to-probable-sarcopenia screening results. Horizontal dashed lines indicate FDR = 0.01. **(E)** Bubble heatmap summarizing ACME, ADE, total effect, and proportion mediated across probable sarcopenia and probable osteosarcopenia proxy for the five prioritized proteins. **(F)** ACME forest plots. **(G)** Proportion mediated forest plots. Candidate labels in panels **(E–G)** denote the prespecified observed-sample classification; final robustness tiers incorporating the age-adjusted screen and full-pipeline bootstrap retain CKB as exploratory (Supplementary Tables S8, S15). ACME, model-based indirect-effect estimate; ADE, average direct effect; FDR, false discovery rate; PSM, propensity score matching.

The matched cumulative-hazard curves remained clearly separated for both osteoporotic fracture and osteoporosis (Figures 2A,B), consistent with the propensity score-matched effect estimates reported above.

In the 2-year baseline lag sensitivity analysis, the fixed group-status estimates remained directionally consistent: HR = 2.40 (95% CI 2.14–2.70; *p* = 6.4 × 10^−49^) for osteoporosis and HR = 3.21 (95% CI 2.32–4.45; *p* = 2.6 × 10^−12^) for osteoporotic fracture. In the additional time-updated Cox analysis, all-cause dementia after diagnosis was associated with osteoporosis across all postdiagnosis person-time (HR = 1.78, 95% CI 1.43–2.20; *p* = 1.5 × 10^−7^) and osteoporotic fracture (HR = 3.68, 95% CI 2.34–5.77; *p* = 1.6 × 10^−8^). Estimates were strongest during the first 0–2 years after diagnosis (osteoporosis HR = 2.89, 95% CI 2.20–3.78; fracture HR = 7.38, 95% CI 4.43–12.27) and attenuated beyond 2 years (osteoporosis HR = 1.09, 95% CI 0.77–1.54; fracture HR = 1.40, 95% CI 0.58–3.41). The early-window estimates were based on 54 osteoporosis and 16 fracture events and may also reflect peri-diagnostic frailty, increased healthcare contact, surveillance, or uncertain temporal ordering (Supplementary Tables S16B–E).

In the additional sociodemographic/lifestyle covariate sensitivity model, all-cause dementia remained associated with incident osteoporosis (HR = 2.17, 95% CI 1.93–2.43; *p* = 1.59 × 10^−39^) and incident osteoporotic fracture (HR = 2.88, 95% CI 2.09–3.98; *p* = 1.47 × 10^−10^). Alzheimer’s disease and vascular dementia showed directionally consistent estimates for the bone outcomes. In the bridge phenotype analysis, all-cause dementia remained associated with probable sarcopenia (OR = 1.31, 95% CI 1.15–1.49; *p* = 6.21 × 10^−5^) in the same sensitivity framework. The subtype-specific probable-sarcopenia estimates were attenuated and did not reach statistical significance in this model (Alzheimer’s disease: OR = 1.05, 95% CI 0.85–1.30, *p* = 0.645; vascular dementia: OR = 1.25, 95% CI 0.97–1.62, *p* = 0.082; Supplementary Table S14).

### 3.4 Dementia was also associated with functional decline and bridge phenotypes

In fully adjusted linear mixed-effects models (Figures 1B,C), all-cause dementia was associated with excess annual grip-strength decline (*β* = −0.317 kg/year; 95% CI − 0.46 to −0.17; *p* = 1.3 × 10^−5^) and accelerated physical-activity decline (*β* = −82.2 MET-min/week/year; 95% CI − 139.8 to −24.6; *p* = 0.005).

Repeat-assessment participation differed substantially by dementia status (Supplementary Table S13A). For grip strength, 37,716 of 150,569 participants without dementia (25.1%) had repeat measures, compared with 163 of 3,223 participants with all-cause dementia (5.1%). For physical activity, 24,568 of 150,517 participants without dementia (16.3%) had repeat measures, compared with 134 of 3,223 participants with all-cause dementia (4.2%). Mortality was higher among participants with all-cause dementia (68.2%) than among participants without dementia (approximately 9.8%). These attendance denominators comprise participants in the Part 1 longitudinal source files with at least one valid outcome measurement and therefore differ by outcome. Among 37,879 participants with repeat grip-strength measurements, 37,869 had complete Model 1 data and contributed 86,251 observations to the LMM; the remaining 10 lacked a usable follow-up time. All 24,702 participants with repeat physical-activity measurements contributed 53,865 observations to the physical-activity LMM (Supplementary Table S13B). Repeat-measure contributors were generally younger, had lower mortality, and had lower dementia prevalence than participants without repeat measures; this comparison was restricted to the 151,751-participant time-to-event cohort, including 37,443 grip-strength and 24,415 physical-activity repeat contributors (Supplementary Table S13C).

Analyses of structural muscle measures (DXA-derived appendicular lean mass and BIA-derived lean mass) in available subsets did not show statistically significant associations with dementia ascertainment status after full covariate adjustment (Supplementary Table S3). This contrast with the grip-strength findings should not be interpreted as evidence that functional decline temporally precedes muscle-mass loss.

Baseline sarcopenia-related phenotypes in the full cohort served as bridge outcomes linking the main epidemiological findings to downstream proteomic analyses (Figure 1D). In the fully adjusted logistic models, all-cause dementia showed higher odds of probable sarcopenia (OR = 1.41, 95% CI 1.23–1.61; *p* = 3.46 × 10^−7^) and probable osteosarcopenia proxy (OR = 1.51, 95% CI 1.23–1.84; *p* = 6.11 × 10^−5^). Vascular dementia also showed elevated odds for both phenotypes after full adjustment, whereas Alzheimer’s disease showed directionally similar but not statistically significant estimates. These bridge analyses showed that the sarcopenia-related outcomes used in the downstream proteomic models were associated with dementia in the full UK Biobank cohort, not only within the Olink subcohort; the corresponding VaD-specific estimates are summarized in Supplementary Table S2.

Where muscle-mass data were available, confirmed sarcopenia was evaluated as an available-subset descriptive check. Confirmed sarcopenia events were sparse: 11 cases among 16,456 participants with available grip and muscle-mass data, including 2 cases among 506 participants with all-cause dementia. Confirmed osteosarcopenia proxy events were even sparser, with 5 total cases and no cases among participants with all-cause dementia. Counts for the confirmed phenotypes are reported in Supplementary Table S10; adjusted models were not fitted because only 11 confirmed sarcopenia and 5 confirmed osteosarcopenia-proxy cases were available.

### 3.5 Prioritized plasma proteins showed candidate indirect-effect signals for the dementia–probable sarcopenia association

In the two-stage protein screening (Figures 2C,D), 600 of 2,923 proteins met the FDR threshold for all-cause dementia in Stage 1. In Stage 2, 300 proteins met the FDR threshold for probable sarcopenia. Of these, 276 also passed Stage 1 (the cross-layer overlap), forming the final shortlist for downstream exploratory model-based mediation analysis. The complete overlap list and enrichment results are provided in Supplementary Table S4. Model-based mediation analyses focused on probable sarcopenia as the primary endpoint, with the broader mediation landscape shown in Figure 2E and individual protein estimates in Figures 2F,G and Table 3.

**Table 3:** Model-based mediation estimates for prioritized plasma proteins in the dementia–probable sarcopenia association.

Protein	Observed-sample class	Sample	Indirect effect		Direct effect	Total effect	Mediation proportion
		*n*	ACME (×10^−3^, 95% CI)	FDR	ADE (95% CI)	Estimate (*p* value)	PM, % (95% CI)
Primary endpoint: probable sarcopenia							
EGFR	Candidate	16,407	1.87 (0.75–3.29)	< 0.001	0.0196 (0.0048–0.0365)	0.0215 (*p* = 0.004)	8.7 (3.5–28.3)
EBI3/IL27	Candidate	16,313	1.23 (0.48–2.26)	< 0.001	0.0198 (0.004–0.038)	0.021 (*p* = 0.010)	5.9 (1.9–19.3)
CKB	Candidate	13,962	0.9 (0.14–1.94)	0.0360	0.0229 (0.0056–0.0422)	0.0238 (*p* = 0.004)	3.8 (0.6–12.7)
GDF15	Exploratory	16,407	1.15 (−0.05–2.5)	0.0875	0.0198 (0.0049–0.0367)	0.021 (*p* = 0.004)	5.5 (−0.3–18.1)
CHGA	Exploratory	13,962	0.41 (−0.14–1.2)	0.1820	0.0229 (0.0058–0.0428)	0.0233 (*p* = 0.010)	1.7 (−0.7–7.5)
Secondary endpoint: probable osteosarcopenia proxy							
EGFR	Candidate	16,407	0.74 (0.23–1.53)	< 0.001	0.0077 (−0.0016–0.018)	0.0084 (*p* = 0.088)	8.8 (−32.4–52.9)
EBI3/IL27	Candidate	16,313	0.6 (0.17–1.24)	< 0.001	0.0078 (−0.0016–0.0175)	0.0084 (*p* = 0.082)	7.2 (−26.1–43.4)
CKB	Candidate	13,962	0.46 (0.05–0.99)	0.0433	0.0079 (−0.0018–0.02)	0.0083 (*p* = 0.114)	5.5 (−42.9–46.3)
GDF15	Exploratory	16,407	0.49 (−0.02–1.11)	0.0875	0.0076 (−0.0007–0.0183)	0.0081 (*p* = 0.062)	6 (−7.4–31.3)
CHGA	Exploratory	13,962	0.36 (−0.16–1.1)	0.1820	0.0079 (−0.0011–0.0195)	0.0082 (*p* = 0.082)	4.4 (−17.8–35.6)

In the age-adjusted Stage 1 sensitivity screen, 2,922 proteins were tested; GLIPR1 was excluded because only 62 complete observations were available, below the prespecified minimum of 1,000. Fifty-one proteins remained significant at BH-FDR < 0.01 and 117 at BH-FDR < 0.05. Among the five prioritized proteins, EGFR (*β* = −0.0412, *p* = 4.17 × 10^−7^, FDR = 6.42 × 10^−5^), EBI3/IL27 (β = 0.0704, *p* = 5.99 × 10^−6^, FDR = 7.00 × 10^−4^), and GDF15 (β = 0.1200, *p* = 2.59 × 10^−8^, FDR = 6.25 × 10^−6^) retained BH-FDR < 0.01 support. CHGA was attenuated to BH-FDR < 0.05, and CKB no longer met BH-FDR < 0.05 in the age-adjusted first-stage screen. We therefore interpreted EGFR, EBI3/IL27, and GDF15 as more robust age-adjusted Stage 1 signals, while CKB and CHGA were treated more cautiously as exploratory downstream signals (Supplementary Table S8).

In the 500-resample selection-aware full-pipeline bootstrap, joint Stage 1–Stage 2 selection frequencies were EGFR 93.8%, EBI3/IL27 65.8%, GDF15 96.6%, CKB 15.0%, CHGA 21.0%. Fixed-target refit ACME estimates (×10^−3^; median [95% percentile interval]) were EGFR 1.78 [0.88 to 3.07], EBI3/IL27 1.19 [0.46 to 2.25], CKB 0.80 [0.10 to 2.00], GDF15 1.05 [−0.04 to 2.27], and CHGA 0.34 [−0.17 to 1.26]. These intervals hold the selected target set fixed and describe effect-estimate variability rather than post-selection validity. Zero-augmented summaries in Supplementary Table S15 combine these estimates with failure to pass joint selection; exact zeros arise by construction when a target is not selected. Taken together, EGFR combined high selection stability with a fixed-target interval excluding zero, and EBI3/IL27 showed moderate selection stability with a fixed-target interval excluding zero. CKB also had a fixed-target interval excluding zero but low joint selection frequency, whereas GDF15 was stably selected but its fixed-target interval included zero. These complementary results support EGFR and EBI3/IL27 as the most consistently supported candidate signals and place CKB, GDF15, and CHGA in an exploratory tier for distinct reasons.

Five triple-supported proteins entered the primary observed-sample exploratory mediation analysis: EGFR, EBI3/IL27, CKB, GDF15, and CHGA. EGFR, EBI3/IL27, and CKB met the prespecified observed-sample criteria of FDR-significant ACME and significant total effects; GDF15 and CHGA did not meet the indirect-effect threshold and were retained as exploratory signals. The age-adjusted and full-pipeline sensitivity analyses were then used to grade the robustness of these observed-sample signals. Here, EBI3/IL27 denotes the Olink assay annotation for the EBI3-containing IL-27 axis.

Among the signals meeting the observed-sample criteria, EGFR showed the largest proportion mediated (PM = 8.7%), with ACME = 0.0019, false discovery rate (FDR) < 0.001, and total-effect *p* = 0.004. EBI3/IL27 showed PM = 5.9% (ACME = 0.0012, FDR < 0.001, total-effect *p* = 0.010), whereas CKB showed PM = 3.8% (ACME = 0.0009, FDR = 0.036, total-effect *p* = 0.004). These mediation proportions were modest, and GDF15 and CHGA showed weaker indirect-effect evidence and were retained as hypothesis-generating signals. In the education-missingness sensitivity analysis (Supplementary Table S7), excluding education from the covariate set yielded directionally consistent estimates for the three prioritized probable sarcopenia candidate signals: EGFR (PM = 8.8%), EBI3/IL27 (PM = 6.0%), and CKB (PM = 3.7%).

The heatmap visualizes the observed-sample indirect-effect estimates for probable sarcopenia; GDF15 and CHGA showed weaker evidence under the prespecified indirect-effect threshold. Robustness across sensitivity analyses is reported in Supplementary Tables S8, S15. For probable osteosarcopenia proxy, mediation directions were generally similar, but the total effects did not reach formal statistical significance in the fully adjusted models. Accordingly, the osteosarcopenia-proxy results were retained as secondary consistency signals, whereas probable sarcopenia remained the primary endpoint.

ACME values are multiplied by 1,000 for readability. The three proteins meeting the observed-sample criteria are labeled Candidate in this table; sensitivity-based robustness tiers are reported separately in Supplementary Tables S8, S15. GDF15 and CHGA are labeled Exploratory for visual and biological context. Bootstrap 95% confidence intervals were derived using the nonparametric percentile method. ACME, model-based indirect-effect estimate reported according to the mediation package terminology; ADE, average direct effect; FDR, false discovery rate; PM, proportion mediated.

### 3.6 Restricted cubic spline analyses supported predominantly linear dose–response patterns

**Figure 3:**
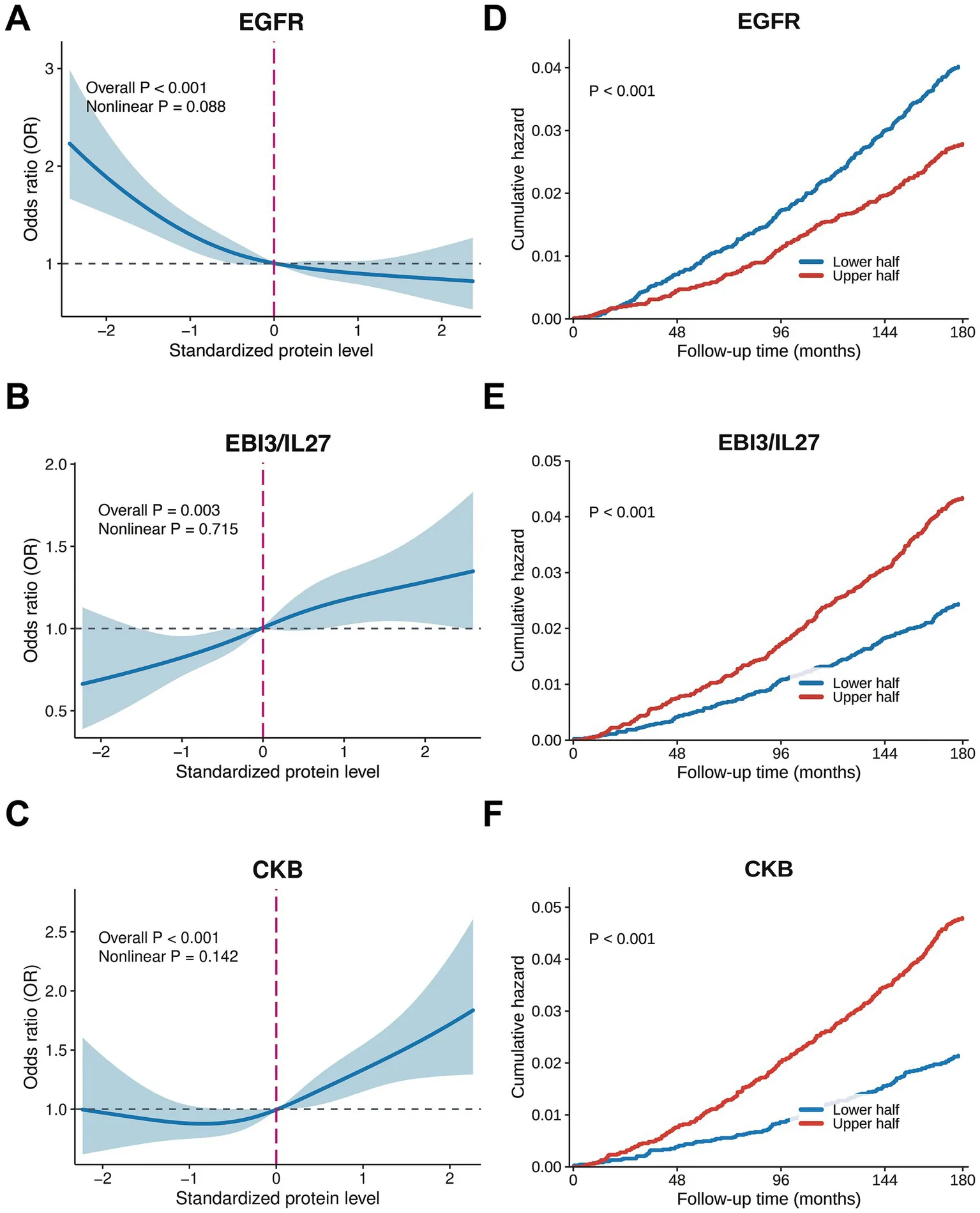
Dose–response and secondary event-based consistency analyses of candidate indirect-effect signals. **(A–C)** Restricted cubic spline curves showing dose–response associations of EGFR, EBI3/IL27, and CKB with probable sarcopenia. Shaded bands represent 95% CIs. **(D–F)** Median-split cumulative hazard curves for incident osteoporosis stratified by EGFR, EBI3/IL27, and CKB levels in the Olink subcohort. *p* values are from log-rank tests; adjusted per-standard-deviation Cox estimates are reported in Supplementary Table S9A. These analyses were conducted within the Olink subcohort and are interpreted as secondary consistency analyses. The three displayed proteins met the prespecified observed-sample indirect-effect criteria; their robustness across sensitivity analyses is reported in Supplementary Tables S8, S15.

Restricted cubic spline analyses demonstrated significant overall dose–response associations between each of the three proteins and probable sarcopenia (Figures 3A–C). The overall association *p* values were < 0.001 for EGFR, 0.003 for EBI3/IL27, and < 0.001 for CKB.

None of the three proteins showed strong evidence of nonlinearity, with *p* values for nonlinearity of 0.088 for EGFR, 0.715 for EBI3/IL27, and 0.142 for CKB. These patterns supported the use of a linear protein specification in the model-based mediation analyses. Among the three proteins, EGFR displayed the clearest gradient; confidence intervals widened at distributional extremes, as expected for sparsely sampled protein levels.

Secondary event-based consistency analyses within the Olink subcohort further showed that several prioritized proteins tracked bone-risk gradients (Figure 3D–F; Supplementary Tables S5, S9A–D). The complete Cox rerun covered 747 unique proteins. Thirteen proteins met BH-FDR < 0.01 in the primary 600-protein family, and 13 met this threshold in the extended 706-protein family. All five prioritized proteins remained FDR-supported in both families across protein-specific models with 468–541 incident osteoporosis events: CHGA (HR = 1.16), CKB (HR = 1.28), EBI3/IL27 (HR = 1.18), EGFR (HR = 0.84), and GDF15 (HR = 1.24). Schoenfeld-residual tests from the same full 13-covariate models gave protein-specific *p* values of 0.103–0.402 and showed no evidence of non-proportional hazards for any of the five protein terms. Global tests indicated departures for EGFR (*p* = 0.017), EBI3/IL27 (*p* = 0.039), and GDF15 (*p* = 0.004), whereas the global *p* values were 0.118 for CHGA and 0.175 for CKB, consistent with time dependence in covariate terms (Supplementary Table S9A). Because the global model tests were not fully satisfied, the reported single-HR protein estimates were interpreted as average associations over follow-up and retained as secondary consistency evidence. Fine-Gray competing-risk models treating death as a competing event showed directionally consistent estimates for the five prioritized proteins (Supplementary Table S9B). For incident osteoporotic fracture in the Olink subcohort, event counts remained sparse (54–65 events across protein-specific models); these targeted fracture protein analyses were treated as underpowered exploratory results.

**Figure 4:**
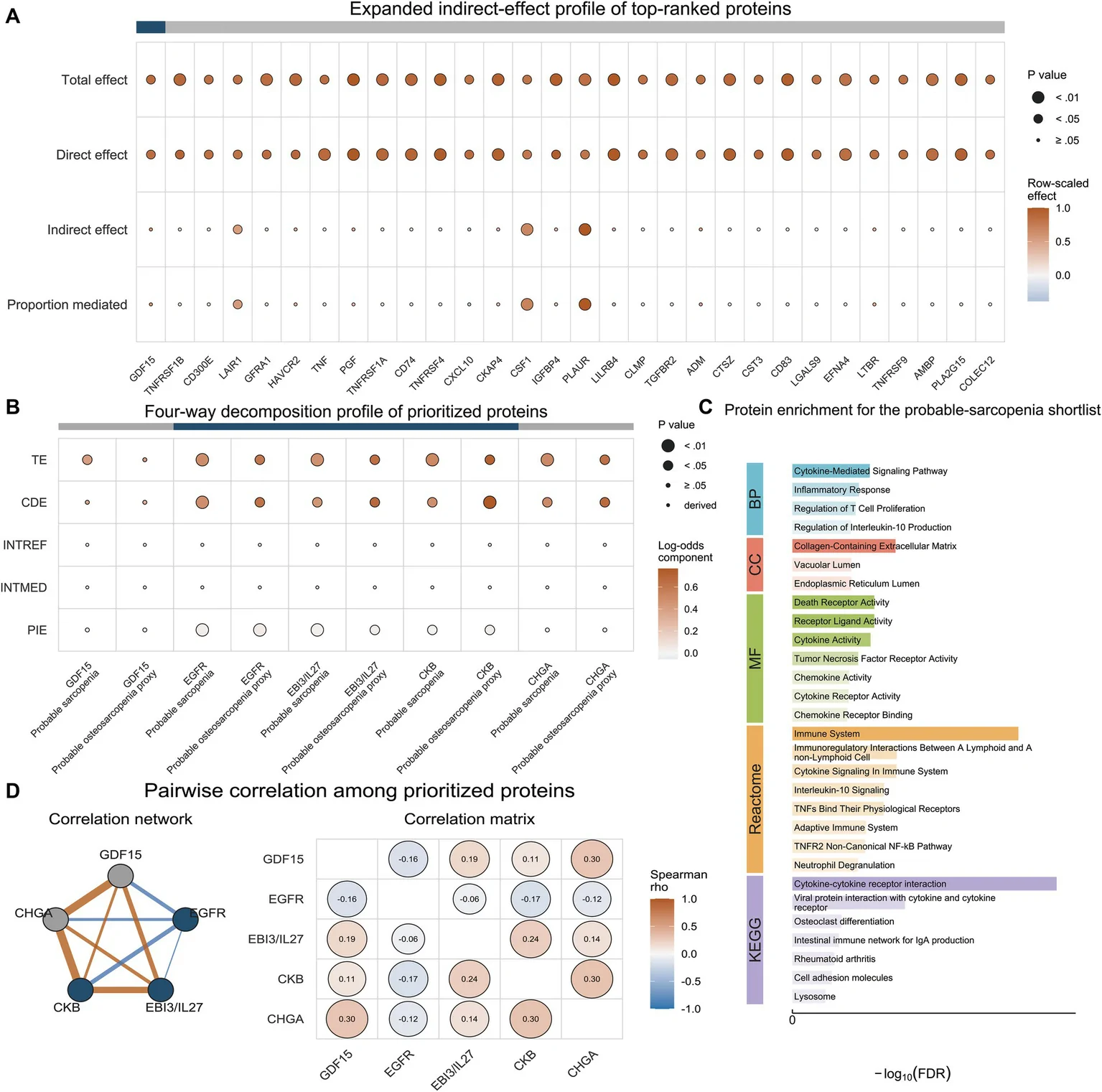
Molecular characterization of prioritized plasma proteins. **(A)** Mediation heatmap of the top 30 ranked proteins from the probable-sarcopenia shortlist, summarizing total, direct, indirect, and proportion-mediated signals. Dot size indicates *p* value; color indicates row-scaled effect magnitude; the top strip distinguishes triple-supported from exploratory proteins. **(B)** Four-way decomposition heatmap for prioritized proteins. Rows denote TE, CDE, INTref, INTmed, and PIE. Dot size indicates significance; color indicates effect magnitude. **(C)** Functional enrichment of the 276-protein probable-sarcopenia shortlist across GO BP, CC, MF, Reactome, and KEGG. Bar length represents −log10(FDR). **(D)** Spearman correlation network among the five prioritized proteins (*n* = 13,588, participants with complete data for all five proteins). Edge width represents |*rho*|; warm colors denote positive correlations and blue denotes negative correlations. All displayed correlations met FDR < 0.001. BP, Biological Process; CC, Cellular Component; MF, Molecular Function; TE, Total effect; CDE, Controlled direct effect; INTref, Reference interaction; INTmed, Mediated interaction; PIE, Pure indirect effect.

Across the broader shortlisted set, a mediation heatmap of the top 30 ranked proteins from the probable sarcopenia shortlist summarized total, direct, indirect, and proportion-mediated signals (Figure 4A). Four-way decomposition using the regmedint framework showed that the pure indirect effect (PIE) was the dominant component for the three statistically supported candidate proteins, with minimal exposure-mediator interaction (Figure 4B), supporting the use of traditional single-mediator estimates as exploratory summaries.

Functional enrichment analysis of the 276-protein probable-sarcopenia shortlist revealed dominant pathways spanning cytokine-mediated signaling, osteoclast differentiation, receptor-ligand activity, and extracellular matrix organization across GO Biological Process, Cellular Component, Molecular Function, Reactome, and KEGG databases (Figure 4C). Spearman correlation analysis among the five prioritized proteins revealed modest inter-protein correlations overall (|rho| range: 0.060–0.304; Figure 4D). The strongest positive correlations were observed between CKB and CHGA (rho = 0.304) and between GDF15 and CHGA (rho = 0.296), whereas EGFR showed negative correlations with all other proteins (rho range: −0.060 to −0.171). This pattern suggests limited correlation among the three prioritized candidate signals.

## 4 Discussion

### 4.1 Principal findings

This large-scale cohort study examined musculoskeletal vulnerability according to algorithmically ascertained dementia status across incident bone fragility, longitudinal functional trajectories, and baseline sarcopenia-related phenotypes. Fixed group-status analyses yielded HRs of 2.31 for osteoporosis and 3.13 for osteoporotic fracture. Time-updated analyses, which aligned risk time to the recorded diagnosis date, yielded overall postdiagnosis HRs of 1.78 and 3.68, respectively, with the strongest estimates during the first 2 years after diagnosis. Participants with recorded all-cause dementia status also showed faster grip-strength decline and higher baseline odds of probable sarcopenia (OR = 1.41).

**Figure 5:**
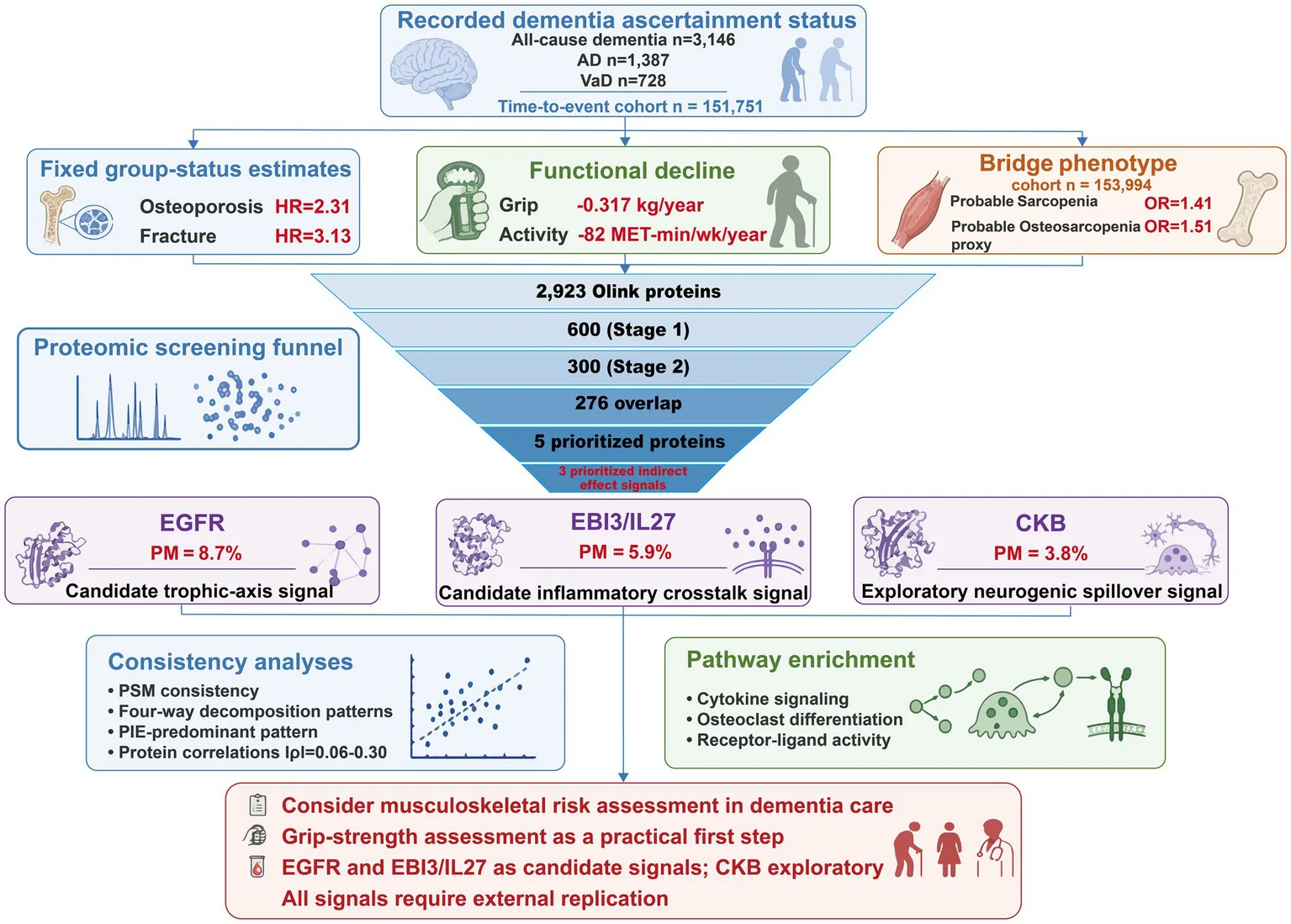
Graphical summary of dementia-associated muscle-bone fragility. The figure integrates the main epidemiological and exploratory proteomic findings. Recorded dementia ascertainment status across linked follow-up is depicted as the grouping variable associated with bone fragility, functional trajectories, and probable sarcopenia-related phenotypes. The displayed bone HRs are fixed group-status estimates. The lower pathway highlights candidate protein signals and dominant enrichment themes. Proteomic signals are interpreted as exploratory model-based indirect-effect findings requiring independent replication. Among the three signals that met the prespecified observed-sample criteria, EGFR and EBI3/IL27 were the most consistently supported across sensitivity analyses. CKB showed lower age-adjusted and bootstrap selection stability and is presented as exploratory. GDF15 was stable in the screening step but did not meet the indirect-effect threshold and is therefore omitted from this pathway summary.

In the fixed group-status analyses, vascular dementia showed the largest point estimates, whereas time-updated subtype estimates were imprecise because relatively few postdiagnosis events accrued. Under the prespecified observed-sample criteria, EGFR, EBI3/IL27, and CKB showed modest candidate indirect-effect signals for the recorded dementia status–probable sarcopenia association, with individual proportions mediated ranging from 3.8 to 8.7%. Across the age-adjusted screen and full-pipeline bootstrap, EGFR and EBI3/IL27 were the most consistently supported, whereas CKB showed lower selection stability and was retained as exploratory. Together, these findings support a cautious muscle-bone fragility framework for dementia-associated musculoskeletal vulnerability (Figure 5).

### 4.2 Mechanistic interpretation

#### 4.2.1 Bone fragility

The magnitude of the bone-fragility associations points to several converging biological mechanisms. First, cognitive impairment reduces mobility and weight-bearing activity, thereby lowering mechanical loading on bone—a primary anabolic stimulus for osteoblast activity. Cerebrovascular and small-vessel disease pathways provide additional context for dementia-related systemic vulnerability (26, 27). Second, dementia-related neuroendocrine disruption—including alterations in hypothalamic–pituitary–adrenal axis signaling and growth hormone/IGF-1 secretion—can impair osteoblast function and accelerate bone resorption (28). Third, neuroinflammatory processes activate peripheral inflammatory cascades that promote osteoclastogenesis through RANKL/OPG imbalance (29).

In the fixed group-status analyses, vascular dementia showed the largest point estimates (HR = 2.60 for osteoporosis; HR = 4.84 for fracture). VaD shares pathophysiological features with cerebrovascular disease, which is linked to accelerated bone loss through vascular calcification and endothelial dysfunction (30, 31), and these mechanisms may converge with age-related impairment in mechanical adaptation and osteoclast–osteoblast coupling (32, 33). The VaD group was the smallest (*n* = 728), and the corresponding time-updated subtype estimates were imprecise.

The fixed group-status subtype pattern is biologically compatible with vascular and inflammatory contributions, but it does not establish etiologic differences between dementia subtypes. In the time-updated analysis, the VaD windows of 0–2 years and more than 2 years contained 7 and 5 osteoporosis events and 3 and 0 fracture events, respectively. These sparse postdiagnosis event counts produced wide confidence intervals, so subtype comparisons remain hypothesis-generating and require larger clinical cohorts.

Despite the strongest epidemiological associations for bone outcomes, the primary binary incident-osteoporosis screen identified 11 proteins at the prespecified FDR threshold (Supplementary Table S6). The time-to-event rerun identified 13 proteins at BH-FDR < 0.01 in the primary 600-protein family and 13 in the extended 706-protein family; the two supported sets differed (Supplementary Tables S9C,D). None entered a supported model-based mediation result for osteoporosis. This pattern may reflect both power constraints and differences in biological pathways. Statistically, the Olink proteomic subcohort (*n* = 16,652) was substantially smaller than the main epidemiological cohort, and incident bone events were relatively rare. Biologically, the pathways linking dementia to bone loss may operate through mechanisms that are not well captured by circulating protein levels.

#### 4.2.2 Muscle function and probable sarcopenia

Recorded dementia status was associated with grip-strength trajectories and probable sarcopenia, whereas lean-mass measures (DXA-ALM and BIA-ALM) did not reach statistical significance. This pattern is compatible with the EWGSOP2 framework, which uses low muscle strength to identify probable sarcopenia and requires reduced muscle quantity for confirmation (18). The available data support grip strength as a practical functional marker; they do not establish temporal precedence between strength loss and structural atrophy (23, 34).

The association between recorded dementia status and baseline probable sarcopenia (OR = 1.41) is consistent with prior cross-sectional reports linking cognitive impairment to low grip strength. Because the phenotype was measured at baseline and most algorithmic dementia dates occurred later, this result is interpreted as a baseline phenotype difference associated with subsequent dementia ascertainment, without assigning temporal direction. Shared endothelial dysfunction, microvascular injury, inflammation, and impaired tissue perfusion remain plausible common contributors (35).

Probable sarcopenia showed a different pattern from bone. Muscle function decline, reflected by grip strength, showed candidate protein signals (EGFR, EBI3/IL27, CKB), possibly because low grip strength-defined probable sarcopenia in dementia reflects inflammatory and trophic signaling disruptions that are detectable in plasma. By contrast, bone loss appears driven more strongly by disuse and endocrine dysregulation, mechanisms less directly captured by circulating proteins after covariate adjustment.

### 4.3 Proteomic mediation signals

The mediation proportions observed for EGFR (PM = 8.7%), EBI3/IL27 (PM = 5.9%), and CKB (PM = 3.8%) were modest. This scale is plausible if the total association is distributed across many pathways, with each protein accounting for only a small fraction. The three proteins were identified through a stringent two-stage FDR-controlled screen of 2,923 proteins. In addition, the restricted cubic spline analyses showed significant dose–response relationships for all three proteins without strong nonlinearity, supporting the linear protein specification used in the exploratory mediation models.

The individual PM estimates for EGFR, EBI3/IL27, and CKB were modest and were derived from separate single-protein models. They should therefore be interpreted as single-protein model-based indirect-effect estimates. These proteins explain only a small fraction of the dementia–probable sarcopenia association. The residual association likely operates through additional behavioral, vascular, inflammatory, neuroendocrine, nutritional, medication-related, and local tissue pathways, including reduced mobility, poor nutrition, antipsychotic or sedative exposure, HPA-axis dysregulation, GH/IGF-1 axis alterations, denervation, satellite cell exhaustion, and marrow adiposity.

Biologically, these three proteins have plausible roles in the broader muscle-bone crosstalk landscape, although their interpretation should remain hypothesis-generating. EGFR signaling is implicated in skeletal muscle regeneration and satellite cell activation (36, 37); its lower circulating levels in dementia patients may reflect impaired peripheral trophic support. EBI3 (Epstein–Barr virus-induced gene 3), the shared soluble subunit of the heterodimeric cytokines IL-27 and IL-35, modulates Th17/Treg balance and inflammatory tone (38–40), both of which are increasingly recognized as regulators of muscle protein catabolism. CKB is a marker of neuronal injury whose elevated plasma levels may indicate blood–brain barrier compromise and secondary neuroinflammatory spillover into the systemic circulation (41, 42). Within the conventional myokine-osteokine framework, EGFR may represent a trophic-axis signal, EBI3/IL27 an inflammatory crosstalk node, and CKB a neurogenic spillover signal.

Among the five triple-supported proteins, GDF15 and CHGA remained exploratory signals in the context of muscle-bone crosstalk. GDF15 is a stress-responsive circulating factor with an established link to skeletal-muscle metabolic stress and systemic energy imbalance, a profile consistent with the neurodegeneration-associated metabolic disruption observed in dementia (43–45). CHGA is a neuroendocrine secretory protein co-released with catecholamines from chromaffin cells and sympathetic neurons (46, 47); its elevated plasma levels in dementia likely reflect heightened autonomic and sympathoadrenal activation, which is mechanistically linked to both accelerated bone resorption and impaired muscle anabolism. The age-adjusted Stage 1 sensitivity screen suggested stronger first-stage support for EGFR, EBI3/IL27, and GDF15 than for CKB and CHGA. Accordingly, CKB and CHGA remain exploratory downstream signals in the current Olink subcohort and warrant cautious interpretation. Longitudinal proteomic studies in clinical dementia cohorts should test whether these proteins track functional decline and broader muscle-bone vulnerability over time.

### 4.4 Limitations

Several limitations warrant consideration. First, the algorithmically derived dementia fields span linked follow-up. Fixed group-status analyses and baseline phenotype/proteomic models therefore characterize associations with dementia ascertainment status rather than effects attributed to dementia already present at baseline. These fixed-status estimates may be affected by temporal exposure misclassification and immortal-time bias. The time-updated Cox analysis improved temporal alignment for bone outcomes by assigning person-time before and on the recorded diagnosis date to the reference state and person-time strictly after that date to the exposed state. Same-day diagnosis-outcome events remained in the reference interval because day-level records could not establish within-day ordering. The bridge phenotype analyses remained cross-sectional, and both proteins and sarcopenia-related outcomes were measured at baseline in the Olink subcohort; the indirect-effect findings therefore remain exploratory statistical decompositions within an observational setting with limited temporal ordering.

Second, the proteomic analyses were conducted within the same Olink subcohort used for screening, prioritization, dose–response modeling, and exploratory indirect-effect analyses. The 500-resample full-pipeline bootstrap separated fixed-target effect-estimate variability from screening stability. Fixed-target intervals remain conditional on the selected target set, whereas zero-augmented summaries combine effect estimates with selection failure. This internal resampling analysis does not establish external reproducibility. The event-based Olink analyses were treated as secondary within-UKB consistency analyses, and independent replication in cohorts with longitudinal proteomics and adjudicated musculoskeletal outcomes remains required. Triangulation using cis-pQTL colocalization and Mendelian randomization would further strengthen causal inference where suitable instruments are available. Protein-specific PH diagnostics from the same full 13-covariate Cox models used for the reported HRs were satisfactory. Global PH tests indicated departures for three osteoporosis models, consistent with time dependence in covariate terms. The corresponding single-HR estimates therefore represent average associations over follow-up and remain secondary consistency signals.

Third, the Olink subcohort was substantially smaller than the main epidemiological cohort and was drawn from a UKB-PPP resource with random baseline and additional non-random selection components. Sampling weights were unavailable in the current analysis-ready files, so proteomic analyses were unweighted. Although included-versus-excluded comparisons showed broadly similar age, sex, BMI, height, and heel eBMD distributions, modest differences in dementia prevalence and mortality indicate possible selection bias (48, 49).

Fourth, repeated-measure analyses were vulnerable to informative attendance and survivor bias. Participants with recorded dementia status were much less likely to contribute repeat grip-strength or physical-activity measures and had substantially higher mortality. Formal loss to follow-up could not be separately identified; we therefore report repeat-assessment attendance, timing of the last observed assessment, and mortality. The LMM estimates reflect trajectories among participants who returned for repeat assessments. Inverse-probability-of-attendance weighting and joint longitudinal-survival modeling are important future sensitivity analyses.

Fifth, residual confounding remains possible. Model 3 included physical activity and several circulating biomarkers that may lie on the dementia-to-musculoskeletal pathway; it was therefore interpreted as an extended conditional association model. The additional sociodemographic/lifestyle sensitivity model supported the main direction of association. The current analysis-ready dataset did not include APOE genotype, medication use, prior falls, or anatomy-specific hip-fracture endpoints. Residual confounding and outcome heterogeneity related to these variables therefore remain possible, and analyses incorporating them are priorities for future work. Retaining education as a missing/unclassified category preserved the analytic sample, and the no-education sensitivity analysis produced similar estimates; residual socioeconomic confounding may nevertheless remain.

Sixth, some outcomes relied on pragmatic proxies. Confirmed sarcopenia assessment was limited by incomplete DXA- or BIA-derived lean-mass data, and confirmed sarcopenia events were very sparse in the available Olink-overlap subset. Low grip strength in cognitively impaired participants may reflect comprehension, motivation, coordination, pain, arthritis, or testing cooperation in addition to muscle capacity. The osteosarcopenia measure should therefore be interpreted as a probable osteosarcopenia proxy. Bone endpoints based on linked hospital ICD-10 codes may also be affected by detection bias because participants with dementia may differ in healthcare contact, hospitalization, diagnostic intensity, and fracture ascertainment.

### 4.5 Clinical implications and conclusions

The time-updated analysis, supported by descriptive fixed group-status results, suggests that osteoporosis and fracture risk assessment may be considered in dementia care, particularly around diagnosis (8, 50). The highest time-updated estimates occurred during the first 2 years after diagnosis and were based on 54 osteoporosis and 16 fracture events. Peri-diagnostic frailty, healthcare contact, surveillance, and uncertain temporal ordering may contribute to this pattern; causal effects and optimal intervention timing remain unresolved. These findings support clinical vigilance, while the value and timing of targeted screening require prospective evaluation. Grip-strength monitoring may provide a practical functional assessment. Subtype-specific recommendations require further evidence because postdiagnosis VaD event counts were sparse.

The identification of EGFR and EBI3/IL27 as the most consistently supported candidate signals, together with the more exploratory CKB signal, suggests circulating markers that warrant further evaluation for musculoskeletal risk stratification in dementia. Longitudinal validation studies in independent clinical dementia cohorts, particularly those with serial proteomic profiling and adjudicated musculoskeletal outcomes, are needed to determine whether protein trajectory changes track functional decline and could serve as monitoring biomarkers. Mechanistically, the trophic (EGFR), inflammatory (EBI3/IL27), and neurogenic (CKB) axes identified here suggest plausible biological links that warrant further experimental and longitudinal evaluation (51).

Muscle-bone vulnerability warrants attention as a clinically relevant component of dementia care. Future longitudinal and interventional studies should align dementia diagnosis dates with musculoskeletal measurements and incorporate adjudicated bone and muscle outcomes.

## Data Availability

Publicly available datasets were analyzed in this study. The data were accessed from the UK Biobank under Application No. 1202100. Access is available to bona fide researchers upon approved application to UK Biobank.

## Supplementary material

SUPPLEMENTARY FIGURE S1 STROBE-style cohort flow. **(A)** Epidemiological cohorts. **(B)** Proteomic subcohort. Participant numbers and reasons for exclusion at each stage, from the local UK Biobank source population (*n* = 501,936) to the Part 1 source cohort (*n* = 154,056), the time-to-event analytic cohort (*n* = 151,751), the phenotype-ready bridge cohort (*n* = 153,994), and the Olink phenotype-ready subcohort (*n* = 16,652). Non-positive follow-up time was defined as follow-up time of zero years or less. The Olink subcohort was analyzed without sampling weights.
